# The Complementary Value of Absolute Coronary Flow in the Assessment of Patients with Ischaemic Heart Disease (the COMPAC-Flow Study)

**DOI:** 10.1038/s44161-022-00091-z

**Published:** 2022-07-04

**Authors:** Louise Aubiniere-Robb, Rebecca Gosling, Daniel J Taylor, Tom Newman, D. Rodney, Hose Ian Halliday, Patricia V Lawford, Andrew J Narracott, Julian P Gunn, Paul D Morris

**Affiliations:** a Mathematical Modelling in Medicine Group, Department of Infection, Immunity and Cardiovascular Disease, University of Sheffield, Sheffield, United Kingdom; b Department of Cardiology, Sheffield Teaching Hospitals NHS Foundation Trust, Sheffield, UK; c Insigneo Institute for In Silico Medicine, University of Sheffield, Sheffield, UK

## Abstract

Fractional flow reserve (FFR) is the current gold-standard invasive assessment of coronary artery disease (CAD). FFR reports coronary blood flow (CBF) as a fraction of a hypothetical and unknown normal value. Although used routinely to diagnose CAD and guide treatment, how accurately FFR predicts actual CBF changes remains unknown. Here we compared fractional CBF with the absolute CBF (aCBF in mL/min), measured with a computational method during standard angiography and pressure-wire assessment, on 203 diseased arteries (143 patients). We found a substantial correlation between the two measurements (r 0.89, Cohen’s Kappa 0.71). Concordance between fractional and absolute CBF reduction was high when FFR was >0.80 (91%), but reduced when FFR was ≤0.80 (81%), 0.70-0.80 (68%) and, particularly 0.75-0.80 (62%). Discordance was associated with coronary microvascular resistance, vessel diameter and mass of myocardium subtended, all factors to which FFR is agnostic. Assessment of aCBF complements FFR, and may be valuable to assess CBF, particularly in cases within the FFR ‘grey-zone’.

Ischaemic heart disease is caused by insufficient coronary blood flow (CBF) to the myocardium. Because CBF cannot be measured directly in the cardiac catheter laboratory (CCL), cardiologists have relied largely upon sensor-tipped wire technologies (pressure, thermistor and Doppler) to derive a number of semi-quantitative indices, each serving as an indirect proxy for estimating CBF restriction.^1^ Of these, pressure-derived fractional flow reserve (FFR) has emerged as the most popular and evidence-based method for guiding revascularisation in intermediate lesions.^2–4^ The value of FFR is that CBF restriction can be quantified, which allows revascularisation to be targeted at lesions that restrict CBF the most. FFR is however, a semi-quantitative assessment of fractional CBF reduction, unable to account for variability in vessel size (diameter), the mass of myocardium subtended and microvascular resistance (MVR), all of which influence absolute CBF. A 15% CBF reduction (FFR 0.85) due to a left main coronary artery stenosis is likely to result in a greater absolute reduction in myocardial blood flow, over a larger myocardial territory, with potentially more serious clinical sequelae, than a 25% CBF reduction (FFR 0.75) resulting from disease in a smaller, more distal vessel. Yet, the opposite may be inferred by the FFR and intervention may therefore, not be targeted at the most physiologically significant lesion. Thus, a method which fully quantifies CBF changes, accounting for these important factors, may be of potential value. Methods for estimating absolute CBF (aCBF) in the CCL have been described.^5–8^ In addition, these methods simultaneously measure absolute microvascular resistance, thus resolving another limitation of FFR. Despite the popularity and importance of FFR in guiding important treatment decisions, the relationship between fractional and absolute CBF changes remains unknown, as does the potential value of these additional measurements when assessing patients for intervention. In this study of absolute CBF, we investigated (i) the relationship between fractional (FFR) and absolute CBF changes, and (ii) concordance and discordance between fractional and absolute CBF changes.

## Results

### Case exclusions and software failures

Two hundred and fifty-six arteries from 169 patients met the inclusion criteria and were recruited. Of these, 20 arteries had insufficient pressure gradient for determining aCBF and 19 had inadequate angiographic views for modelling, leaving 217 suitable cases. Of these, seven failed to mesh and seven failed to converge during numerical simulation. 203 arteries from 143 patients were included in the final analysis. Therefore, of those cases meeting the clinical inclusion criteria, the CFD method computed physiological results successfully in 93.5% of cases. A software problem resulted in vessel diameter data being unavailable in 28 cases.

### Baseline patient and vessel characteristics

The baseline demographic and clinical characteristics of all 143 patients are shown in Table 1. Artery- and lesion-specific characteristics are summarised in Table 2. Overall, 51% of arteries underwent PCI and 49% were treated medically. Ninety-three percent of arteries with a positive FFR (≤0.80) underwent PCI and seven percent were treated with medical therapy, whereas two percent of cases with a negative FFR (>0.80) underwent PCI and 98% were treated medically. Median hyperaemic aCBF, coronary flow reserve (CFR) and microvascular resistance was 85.2 (63.5–116.3 mL/min), 1.62 (1.32-2.04) and 0.71 (0.52-0.98 mmHg.min/mL). The aCBF was higher in the LMS, LAD and RCA cases (95.6, 81.6 and 98.8 mL/min) than the LCX, diagonal and OM cases (78.1, 79.9, 56.7 mL/min), but these differences did not reach statistical significance.

### Reduction in absolute coronary blood flow

The median reduction in aCBF (from hypothetical normal, caused by epicardial disease) was 20.6 (12.4–38.3) mL/min. FFR positive cases were associated with a greater reduction in aCBF than FFR negative cases (34.6 vs 11.6 mL/min, P<0.0001). The threshold for significance for reduction in aCBF was determined as a reduction in aCBF of ≥23 mL/min. Reduction in aCBF was numerically but statistically non-significantly higher in LMS, LAD and RCA cases (median values; 45.7, 21.1 and 23.7 mL/min) compared with LCX, diagonal and OM branch cases (20.8, 17.8 and 12.8 mL/min).

### Relationship between fractional and absolute flow reduction

**Figure 1:**
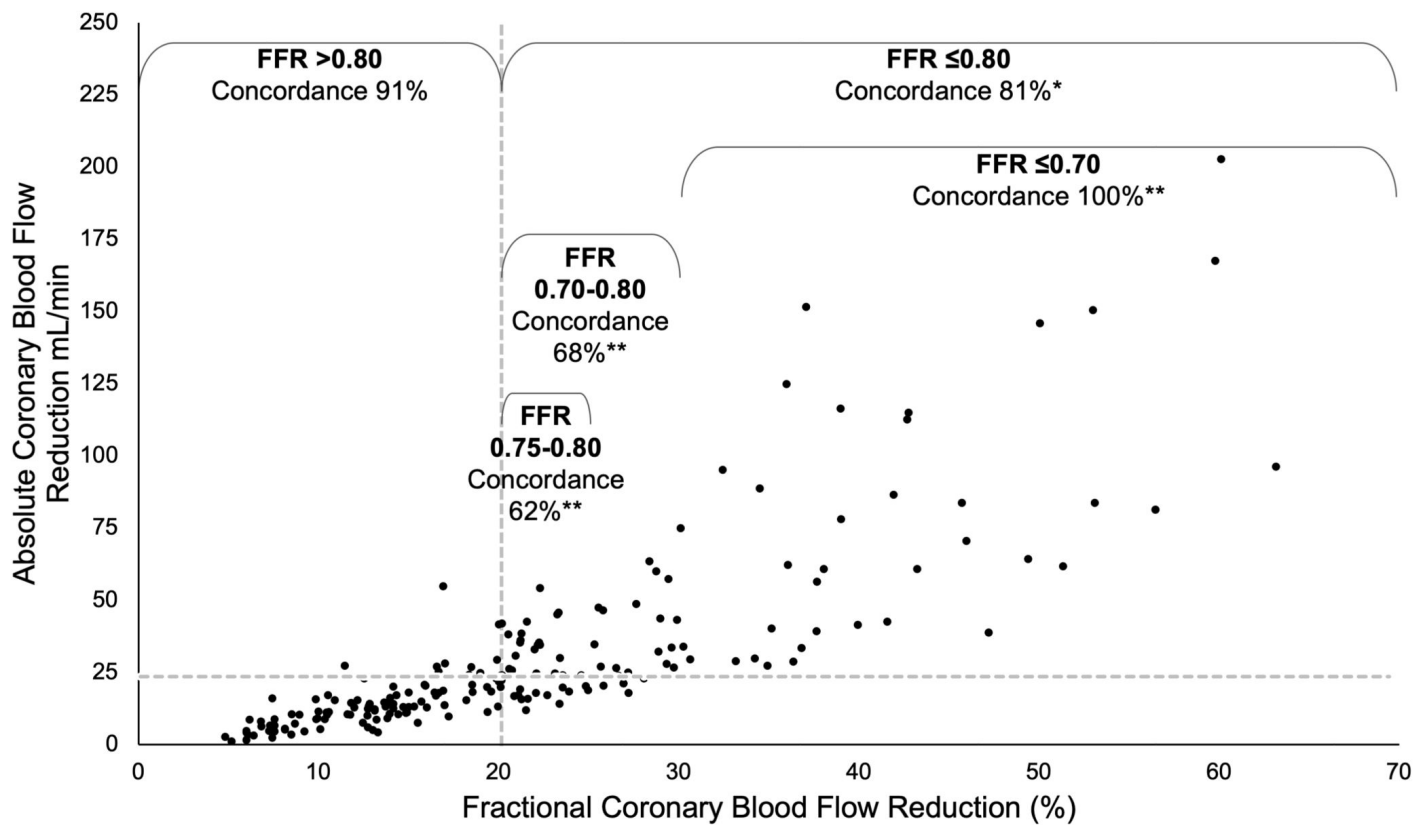
The relationship between fractional and absolute coronary blood flow reduction. Concordance between fractional and absolute CBF reduction is indicated by the labels according to the FFR range. Concordance means significant FFR (≤0.80) and significant reduction in aCBF (≥23 mL/min), or insignificant FFR (>0.80) and insignificant reduction in aCBF (<23 mL/min). The horizontal and leftward vertical dashed lines reflect the thresholds for physiological significance for FFR and aCBF. The asterisks indicate statistical significance for differences in concordance (z scores) compared with the FFR>0.80 group at, the P<0.05* and P<0.01** level (exact P values [2 tailed] are 0.021 for the ≤0.80 group, <0.001 for the 0.70-0.80 and 0.75-0.80 groups and 0.02 for the ≤0.70 group, unadjusted for multiple comparisons). No replicates.

Fractional CBF reduction (measured by FFR) was plotted against aCBF reduction (measured by the CFD method). Despite a strong correlation between fractional and absolute CBF reduction (*r* 0.89, 0.86-0.92, P<0.001) there was variability between individual cases (Figure 1). When the cases were divided into those with significant or non-significant CBF reduction according to FFR (fractional reduction) and according to aCBF (absolute reduction), the Cohen’s Kappa statistic was 0.71 indicating a substantial agreement between the two parameters.

### Concordance between fractional and absolute flow reduction

All 203 cases were categorised as physiologically concordant (significant FFR and significant aCBF reduction, or non-significant FFR and non-significant aCBF reduction) and discordant. Discordance between fractional and absolute CBF reduction was low in FFR negative (FFR >0.80) cases (9%). Discordance was significantly higher in FFR positive cases (19%, P <0.05), higher still when FFR was between 0.70-0.80 (32%, P<0.01) and highest when FFR was in the 0.75-0.80 ‘grey-zone’ (38%, P<0.01). Discordance was 0% in cases with FFR<0.70 (Figure 1).

### Factors affecting discordance

**Figure 2:**
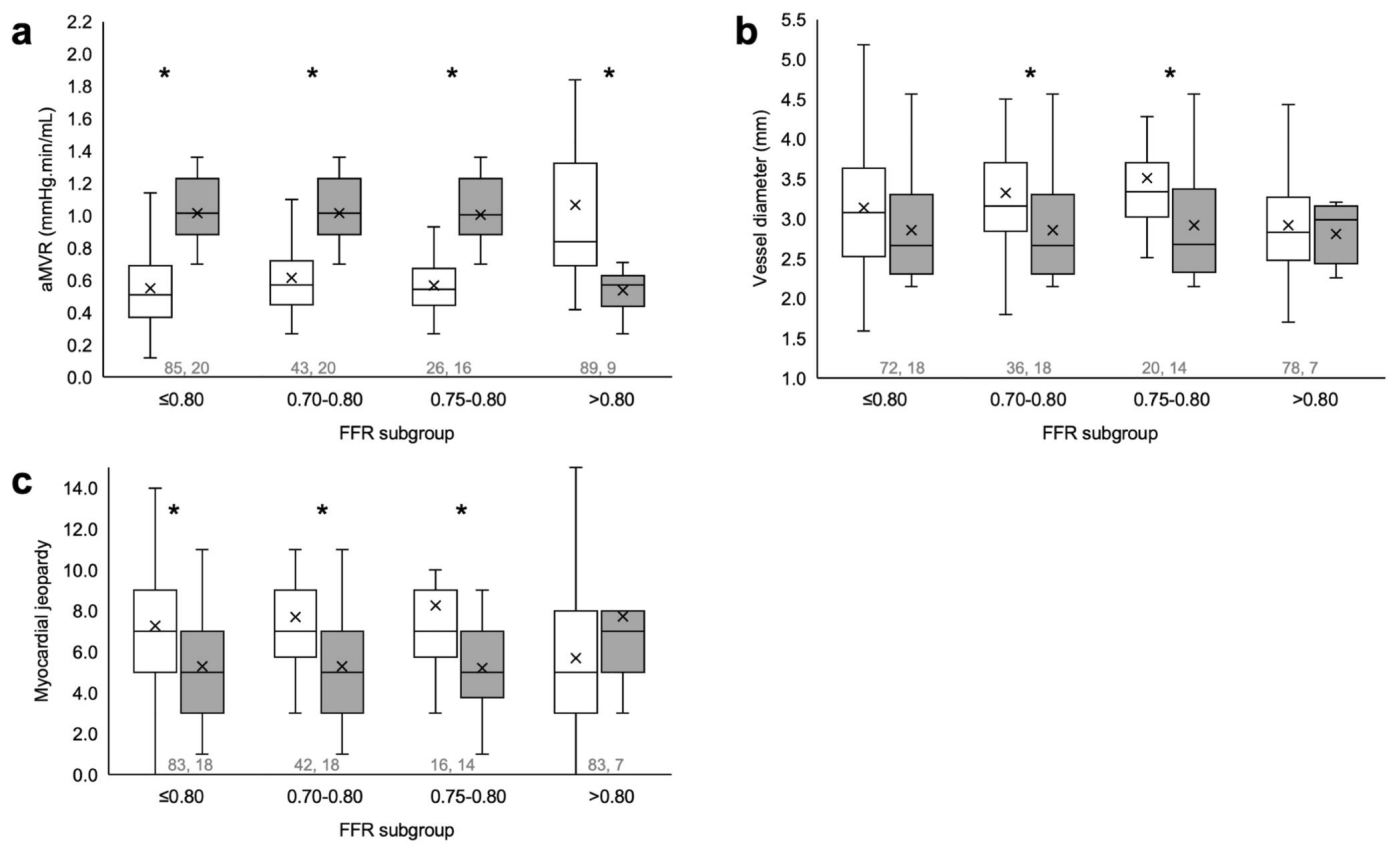
Box and whisker plots demonstrating the difference between concordant (white boxes) and discordant (shaded boxes) cases in each FFR subgroup on the basis of absolute microvascular resistance (panel A), vessel diameter (panel B) and myocardial jeopardy (panel C). The direction of these differences are associated with discordance between absolute and fractional coronary blood flow changes. Concordance was 100% in the FFR <0.70 group; hence the absence of a plot for this subgroup. The box defines the interquartile range, the line and x indicate the median and mean, and the bars represent variability outside the upper and lower quartiles excluding outliers. Asterisks indicate statistical significance at the P≤0.05 level (P values for panel **a** are all <0.001, for panel **b** are 0.03 and 0.02, and for panel **c** are 0.018, 0.019 and 0.021 respectively, calculated in Rstudio (two-sided Mann Whitney U) with no adjustment for multiple comparisons). The numbers in grey indicate N for each group, in each comparison (no replicates).

When comparing the fractional and absolute reduction in CBF, differences in coronary MVR, vessel diameter and myocardial jeopardy were identified that were associated with discordance between these two measures (Figure 2). In cases with FFR ≤0.80, aMVR was significantly higher (causing reduced aCBF) in discordant cases than concordant cases (1.02 vs 0.51 mmHg.min/mL, P<0.01). The same was true of the FFR 0.70-0.80 and 0.75-0.80 groups (1.02 vs 0.57 and 1.00 vs 0.55 mmHg.min/mL, respectively, P<0.01 for both comparisons). In cases with FFR >0.80, aMVR was significantly lower (causing higher CBF) in discordant cases than concordant cases (0.57 vs 0.84 mmHg.min/mL, P<0.01) (causing increased CBF). Myocardial jeopardy was consistently and significantly lower in discordant cases than concordant cases in all cases with FFR ≤0.80 (7.28 vs 5.28, P<0.05), in those with FFR 0.70-0.80 (7.69 vs 5.28, P<0.05) and in those with FFR 0.75-0.80 (8.27 vs 5.21, P<0.05). In cases with FFR >0.80 myocardial jeopardy was numerically higher in discordant cases but this difference did not reach statistical significance (7.71 vs 5.69, P=NS). Vessel diameter was significantly greater in concordant than discordant in cases with FFR 0.70-0.80 and in those with FFR 0.75-0.80 (3.33 vs 2.85 mm P<0.05 and 3.51 vs 2.92 mm P<0.05). In cases with FFR >0.80 and in those with FFR ≤0.80, there was no significant difference in vessel reference diameter between concordant and discordant cases (2.92 vs 2.81 mm, P=NS, 3.14 vs 2.85 mm, P=NS). Thus, heterogeneity in aMVR, myocardial jeopardy and vessel size appeared to contribute to discordance between fractional and absolute CBF reduction.

## Discussion

The main findings of this study were that there was a substantial correlation between fractional and absolute flow reduction, but that individual case variation resulted in discordance in 9% of cases with a physiologically non-significant FFR and 19% of those with a physiologically significant FFR. Discordance was greatest in cases within the FFR ‘grey-zone’ where it reached 38%. Discordance between fractional and absolute CBF reduction appeared to be associated with variability in three related factors; coronary MVR, myocardial jeopardy (a marker of mass of myocardium subtended) and vessel size.

The ability to fully quantify aCBF changes may extend the benefits currently provided by FFR. It would be reasonable to infer that a lesion that reduces CBF by 50 mL/min is more likely to cause symptoms and clinical sequalae than a lesion that reduces CBF by 10 mL/min. FFR is effectively normalised for vessel size, but is agnostic to differences in absolute flow. Whether symptoms and clinic sequalae are better predicted by the absolute or the fractional flow reduction remains unknown. This was not the subject of this study. Rather, this study demonstrated that there was variability between absolute and fractional CBF reduction. In the current study, disparity between fractional and absolute CBF reduction was associated with changes in three related factors; vessel size (diameter), myocardial resistance and the mass of myocardium subtended by that artery. FFR does not account for these parameters, all of which are important determinants of CBF. Instead, in real-world practice the operator assesses these factors automatically by ‘eye-balling’ the angiogram. Similar relationships have been demonstrated previously with CFR and the index of microcirculatory resistance (IMR). Variability in IMR accounts for discordance between FFR and Doppler-derived CFR.^14,15^

The novel method also predicted absolute coronary microvascular resistance (aMVR) which may be advantageous for several reasons. First, naturally occurring and pathological variability in aMVR is known to explain discordance between pressure- and flow-based intracoronary assessment.^15,16^ Second, MVR assessment is helpful in the diagnosis of coronary microvascular disease and is now supported by a 2A recommendation in the European Society of Cardiology guidelines.^17^ Microvascular disease responds to medical therapy, but is rarely diagnosed in the catheter laboratory because standard tests like FFR cannot identify or measure it.^18^ Third, it has been suggested that concomitant microvascular dysfunction may be one reason why 20% of patients do not achieve full symptomatic relief with PCI, even when FFR-guided.^4^ It is therefore interesting that the rate of discordance in FFR ‘positive’ patients in our study was similar at 19%.

FFR was least reliable at predicting aCBF changes in cases with FFR 0.70-0.80 (68% concordance) and poorest in the range 0.75-0.80 (62% concordance). This is interesting because this FFR range corresponds with what has become known as the FFR ‘grey-zone’. The DEFER trial originally proposed FFR >0.75 as the threshold for deferring intervention in favour of medical therapy^19^ whereas the seminal FAME and FAME-2 trials supported a threshold of FFR >0.80.^20,21^ The higher threshold increases the sensitivity for detecting flow limiting lesions, but comes at the expense of reducing specificity. It is therefore, interesting that the current study identified those with an FFR in the same range as being most discordant with aCBF.

The values of aCBF reduction are lower than those reported for the continuous infusion thermodilution (CIT) method.^22^ There are two main reasons for this. First, our population had haemodynamically significant epicardial coronary artery disease, whereas data supporting the CIT method is largely derived from those with unobstructed coronary arteries. Second, the novel CFD method determines the coronary outlet flow, whereas CIT predicts inlet flow; the former being much greater due to the loss of flow to side branches, particularly in the LAD which supplies multiple branches to the lateral wall and septum. Outlet flow is a lesion-specific parameter which allows the reduction in CBF, due to proximal disease to be derived, which may be useful when predicting the physiological value of PCI and making revascularisation decisions. Finally, the two methods are distinct and a systematic or methodological influence cannot be excluded.

The methods used in this study do not compete with, or invalidate FFR. Quite the opposite is true. FFR predicts CBF as a fraction of an hypothetical and unknown value. The novel method translates this into a value, converting a semi-quantitative into a fully-quantitative measurement. Moreover, the novel method requires the data acquired during FFR and the mathematics used in its derivation. In this way, the two methods may be complementary. Given that agreement between FFR-predicted flow reduction and aCBF reduction was high in cases where FFR was >0.80 and perfect when FFR was ≤0.70, a hybrid approach to coronary assessment may have value, assessment of aCBF only being required if the FFR value lay in the range 0.70-0.80. If this approach were applied to the current dataset, FFR alone would be sufficient in 65% of cases and additional assessment of aCBF would be required in 35%.

The study was observational, and so the results are hypothesis generating. The clinical benefits of assessing CBF reduction in absolute terms are yet to be proven. The application of the proposed hybrid assessment is hypothetical. Currently, the CFD method does not account for flow to side branches which underestimates flow in the proximal vessel. The novel method awaits in vivo validation but has been tested in vitro, in unbranched phantom models. It requires a pressure gradient of at least 4 mmHg which roughly equates to a translesional pressure ratio of 0.95. The latter means that, at the current stage of development, the method cannot be used in clearly unobstructed epicardial coronary arteries which is a limitation in patients under investigation for ischaemia with no obstructive coronary disease. Due to an error in the software, vessel diameter data were unavailable in 28 cases (13.7%).

### Conclusions

In patients with coronary artery disease, there was a substantial correlation between (pressure-derived) fractional and (computed) absolute coronary blood flow reduction. Concordance between fractional and absolute flow reduction was high when FFR >0.80 and <0.70 but was poorer when 0.70-0.80, and poorest in the 0.75-0.85 ‘grey-zone’ range. Assessment of absolute CBF reduction may complement FFR and extend its benefits in selected cases.

## Methods

### Patient inclusion and exclusion criteria

The study was approved by the South Yorkshire Health Research Authority Regional Ethics Committee (16/NW/0897 and 08/H1308/193). Patients were eligible for inclusion if they were being investigated for chest pain by invasive coronary angiography and pressure wire assessment, were aged 18 years or older and provided informed consent where appropriate. Participants were not compensated for inclusion and were recruited from elective and inpatient cardiac catheter lists at Sheffield Teaching Hospitals NHS Foundation Trust. Only cases suitable for pressure-wire assessment were included and so results may not be representative in other cases. Patients with chronic and acute coronary syndromes were included but, in acute cases only non-culprit arteries were studied. Patients with ST-segment elevation myocardial infarction within 60 days, contraindication to adenosine or contrast media, previous coronary artery bypass surgery, chronic total occlusion, severe valvular disease, inability to consent or without angiographically significant coronary artery disease were excluded. The clinical data of all patients are reported in Supplementary Dataset 1.

### Angiographic and pressure wire data collection

Invasive coronary angiography was performed according to standard clinical protocols. Operators were encouraged to optimise target artery opacification, acquire clear views of the stenosis region and minimise vessel overlap and panning to facilitate arterial reconstruction.^9^ A panel of three cardiologists, independent of physiological simulation, evaluated each angiogram to assess the reconstruction. Each panel member had to be satisfied for a case to be included. The panel also assessed global and vessel-specific myocardial ischaemic jeopardy index and the percentage lesion stenosis for all cases. The myocardial jeopardy index is a lesion-specific measure of the number of myocardial segments jeopardized by a stenosis, and is therefore a marker of the mass of myocardium subtended.^10^ Pressure wire assessment also proceeded according to standard protocols with translesional pressure measurements acquired under baseline and hyperaemic conditions. Hyperemia was induced with an intravenous infusion of adenosine (140 mcg/kg/min).^11^ The method for deriving aCBF (detailed below) utilises data from angiography and an 0.014” pressure wire. In this study, the PressureWire X (Abbott Laboratories) and PrimeWire Prestige (Philips Volcano) were used. Any lesion with FFR ≤0.80 was regarded as physiologically significant and treated accordingly. FFR reports CBF as the fraction of CBF that remains, in an epicardial coronary artery, relative to an hypothetical normal value for that artery, under maximal flow conditions. Fractional (percentage) reduction in flow was therefore calculated as 1-FFR, multiplied by 100. The hemodynamic data are included in Supplementary Dataset 2.

### Deriving absolute coronary flow and absolute coronary microvascular resistance

Absolute coronary blood flow (aCBF) was computed by the computational fluid dynamics (CFD) model. Three-dimensional coronary arterial anatomy was reconstructed from two angiographic projections ≥30° apart, acquired during ECG-gated end diastole, using an epipolar line transaction method.^12^ The panel of cardiologists assessed all arterial reconstructions and models against the angiographic images to ensure each reconstructed model was based on the artery in question. The reconstructed arterial volume was discretised, measured pressures were applied as boundary conditions, and a numerical simulation based upon solving the 3D, incompressible Navier-Stokes and continuity equations was performed. The principal model output was a calculation of aCBF, distal to the epicardial stenosis/es, at the location of the pressure wire transducer. A detailed description of the method has been published.^5^ Only cases with FFR ≤0.95 or ≥4 mmHg translesional pressure gradient were included because an insufficient pressure gradient is associated with increased model error. By combining measurements of aCBF and pressure, absolute microvascular resistance (aMVR) and the reduction aCBF due to epicardial disease were also calculated. Absolute microvascular resistance (mmHg.min/mL) was calculated according to the hydraulic equivalent of Ohm’s law, as the ratio of distal pressure (P_d_) and aCBF $\left(\frac{{\text{P}}_{\text{d}}}{\text{aCBF}}\right)$. The aCBF in the hypothetical non-stenosed artery was calculated as the ratio of aCBF and FFR $\left(\frac{\text{aCBF}}{\text{FFR}}\right)$, and the reduction in aCBF due to epicardial coronary disease was calculated as difference between this and aCBF. These physiological parameters were calculated on a vessel-specific basis under baseline and hyperaemic conditions.

The primary outcome was to assess the relationship (correlation, concordance and discordance) between fractional (FFR) CBF reduction and absolute CBF reduction in a population of patients with coronary artery disease. Subgroup analysis was also performed according to clinically relevant ranges of FFR (≤0.80, >0.80, 0.70-0.80, 0.75-0.80 and ≤0.70). Simulations were performed on a Dell Precision T5600 computer (Intel Xeon E5 2560, 2GHz processor, 32GB RAM) or a Dell Precision 5540 mobile workstation (Intel Core i9-9980HK processor, 32GB RAM).

### Statistical Analysis

Statistical analysis was performed in RStudio 1.2.1335 (R, v3.6.1) and in MS Excel (16.16.27). Normality of distribution was assessed with histograms, QQ plots and the Shapiro-Wilk test. Parametric and non-parametric continuous data are presented as mean (±SD) and median (IQR) respectively. Wilcoxon Signed Rank and Mann-Whitney U tests were used to compare paired and unpaired grouped data. Levene’s test for homoscedasticity was used and we found evidence of heteroscedasticity. Therefore, the Kruskal-Wallis test was used to compare groups of data (rather than a standard ANOVA). Proportions were compared by calculating the z-score. To determine the threshold for significance for aCBF reduction, a power regression model (y = a·x^b^) was applied to aCBF and fractional CBF reduction (1-FFR) which were plotted against each other, at a point equivalent to FFR=0.80. Correlation between aCBF and percentage flow reduction was assessed with Pearson’s correlation coefficient (*r*) after log transformation for continuous data and with Cohen’s Kappa correlation coefficient (k) for categorical data (concordance and discordance) which adjusts for agreement expected by chance and is a number between −1.0 and 1.0 with values of 0, 0.10–0.20, 0.21–0.40, 0.41–0.60, 0.61–0.80, 0.81–0.90 and 1.0 indicating none (equivalent to chance), slight, fair, moderate, substantial, near-perfect and perfect agreement, respectively.^13^ Negative values indicate agreement worse than that expected by chance. To detect a 15% reduction in concordance (α 0.05) with 80% power, we required around 50 cases in each subgroup. We aimed to include 200 cases in total, to ensure ≥50 in the major sub-group comparisons (FFR positive vs negative and vs 0.70-0.80). P values were not corrected for type 1 error (alpha) for multiple comparisons.

## Supplementary Material

Dataset 1

Dataset 2

Source data for figure 1

Source data for figure 2

## Figures and Tables

**Table 1:** Baseline patient characteristics. SD; standard deviation, IQR; interquartile range, MI; myocardial infarction, COPD; chronic obstructive pulmonary disease, LVEF; left ventricular ejection fraction, eGFR; estimated glomerular filtration rate.

Characteristic	Study Population (n=143) n (%), mean ±SD, or median (IQR)
Demographic characteristics	
Mean age (years)	65 ±10
Male	108 (75.5)
White British	128 (89.5)
Current tobacco use	21 (14.7)
Previous tobacco use	70 (49)
Common Diagnoses	
Previous MI	36 (25.2)
Hypertension	93 (65)
Hyperlipidaemia	108 (75.5)
Diabetes mellitus	37 (25.9)
Obesity	39 (27.3)
COPD	10 (7)
Asthma	4 (2.8)
Peripheral vascular disease	4 (2.8)
Atrial fibrillation	5 (3.5)
Valvular heart disease	7 (5)
LVEF<50%	29 (20.3)
Haematology and Biochemistry	
eGFR (mL/min/1.73m^2^)	82 (66-90)
Haemoglobin (g/dL)	141.3 ± 14.6

**Table 2:** Vessel and lesion characteristics. FFR; fractional flow reserve, IQR; interquartile range. Ischaemic burden indicates the vessel-specific myocardial jeopardy score.

Artery	N (%)	FFR Median (IQR)	FFR ≤0.80 N (%)	Average % stenosis	Ischaemic burden (myocardial jeopardy)
Left anterior descending	103 (50.7)	0.79 (0.71-0.85)	58 (56.3)	59%	6.5
Right coronary	45 (22.2)	0.80 (0.63-0.88)	25 (55.6)	66%	7.6
Left circumflex	26 (12.8)	0.80 (0.71-0.87)	14 (53.8)	64%	4.6
Diagonal	17 (8.4)	0.82 (0.79-0.86)	7 (41.2)	53%	5.1
Obtuse marginal	7 (3.4)	0.87 (0.79-0.93)	2 (28.6)	58%	3.7
Left main	5 (2.5)	0.76 (0.65-0.79)	3 (60)	63%	13.4
Total	203	0.80 (0.72-0.87)	109	61%	6.4

## Data Availability

All the data supporting the findings in this research letter are provided as source data and supplementary information to this manuscript.
